# Identification, Pathogenicity, and Reverse Genetics System Construction of a Pseudorabies Virus Isolate from Pigs in China

**DOI:** 10.3390/vetsci12060519

**Published:** 2025-05-26

**Authors:** Mo Zhou, Haiyang Liang, Nannan Nie, Li Zhang, Rui Zhu, Shinuo Cao, Shanyuan Zhu

**Affiliations:** 1 Jiangsu Key Laboratory for High-Tech Research and Development of Veterinary Biopharmaceuticals, Jiangsu Agri-Animal Husbandry Vocational College, Taizhou 225300, China; mo_zhou@jsahvc.edu.cn (M.Z.); lzhang@jsahvc.edu.cn (L.Z.); rzhu2021@jsahvc.edu.cn (R.Z.); 2 Ningbo Sansheng Biological Technology Co., Ltd., Ningbo 315000, China; lianghaiyang@bio-ss.net; 3 MOA Key Laboratory of Animal Bacteriology, MOE Joint International Research Laboratory of Animal Health and Food Safety, College of Veterinary Medicine, Nanjing Agricultural University, Nanjing 210095, China; 2023807111@stu.njau.edu.cn

**Keywords:** pseudorabies virus, fosmid library, genetic manipulation platform, recombinant PRV

## Abstract

Pseudorabies virus (PRV) is a highly contagious pathogen in swine that can cross species barriers and infect other mammals, including humans. Given the potential for interspecies transmission and its threat to public health, understanding the molecular biology of PRV strains is essential for developing effective control measures and preparing for future pandemics. In this study, a novel PRV strain, PRV-HL-2021, was isolated from an outbreak in Heilongjiang Province, China. The viral genome was used to establish a reverse genetics system based on a fosmid library of the PRV-HL-2021 genome. This system facilitated the creation of recombinant PRV, including one expressing EGFP and another with deletions in the *US9, gI,* and *gE* genes. PRV-HL-2021 was found to be highly lethal to mice *in vivo*. The recombinant PRV strains, such as rPRV-US9-EGFP and rPRV-del*gI/gE/US9*, exhibited growth characteristics similar to the parental PRV-HL-2021 strain. The isolation and characterization of PRV-HL-2021 contribute to a better understanding of the genetic diversity of PRV strains. The developed reverse genetics system provides valuable tools for investigating viral functions, creating genetically modified PRV strains, and advancing the development of safer vaccines. These findings will enhance strategies for controlling PRV outbreaks and mitigating its impact on both animal and public health.

## Simple Summary

Pseudorabies virus (PRV) is a highly contagious virus that primarily affects pigs but can also infect other animals, including humans. In this study, a novel PRV strain, PRV-HL-2021, was isolated from an outbreak in Heilongjiang, China. This strain was found to be highly lethal to mice, highlighting its pathogenicity. A reverse genetics system was established using a fosmid library of PRV-HL-2021’s genome, enabling the creation of recombinant PRV strains, including one expressing enhanced green fluorescent protein (EGFP) and another with deletions in key viral genes (*US9*, *gI*, and *gE*). These recombinant strains were used to study viral replication, pathogenesis, and gene functions. The successful isolation of PRV-HL-2021 and the development of this reverse genetics system provide valuable tools for understanding PRV’s genetic diversity and for developing safer vaccines and antiviral strategies. These advancements will help control PRV outbreaks in pigs and reduce the risk of transmission to humans and other animals.

## 1. Introduction

Pseudorabies (PR), also known as Aujeszky’s disease, is a highly contagious viral infection that poses significant economic threats to the global swine industry [1,2]. It is caused by the pseudorabies virus (PRV) and primarily affects pigs, resulting in severe clinical symptoms such as high mortality rates in piglets, reproductive failures in sows, and growth retardation in fattening pigs [3]. PRV is capable of infecting various animal species such as pigs, wild boars, bears, and ruminants like cattle, sheep, and goats, as well as carnivores like foxes and minks, and rodents. Among all susceptible species, pigs and wild boars uniquely act as natural hosts and reservoirs of the virus [4,5]. The clinical signs in infected pigs include fatal neurological disorders in newborn piglets [6,7], respiratory issues and growth inhibition in growing and fattening pigs, and reproductive failures in sows [8,9]. Recently, cases of acute encephalitis in humans in China, characterized by respiratory and neurological symptoms, have been linked to an emerging PRV variant in the region. This highlights the potential for PRV transmission from pigs to humans and underscores the need for increased global awareness of the disease [10,11,12]. Therefore, investigating circulating PRV strains is essential for both public health and veterinary well-being [12].

PRV is a virus with double-stranded DNA, classified under the Herpesviridae family and the Alphaherpesvirinae subfamily [13,14]. The virus has a large genome of approximately 130–150 kb [15,16], which poses challenges for developing genetically modified viral strains for research and vaccine production [14,17,18]. Traditional methods for constructing recombinant PRV strains involve homologous recombination, which is labor-intensive and often results in low success rates [19]. Recent advances, such as CRISPR/Cas9 technology, have improved the efficiency of virus engineering but still require lengthy processes such as plaque purification and several rounds of viral passaging [20,21,22,23]. Additionally, bacterial artificial chromosomes (BACs) have been used to generate infectious clones of herpesviruses; however, this approach is time-consuming and technically challenging. In contrast, fosmid-based systems have proven to be a more efficient and practical choice for assembling infectious clones of large DNA viruses [13]. Fosmids offer advantages such as easier manipulation, higher cloning capacity, and the ability to handle large genomic fragments, making them ideal for PRV reverse genetics studies. 

In this study, we isolated and characterized a recently emerged PRV strain from a swine outbreak in Heilongjiang Province, China, and evaluated its pathogenicity in mice. To gain deeper insights into PRV biology and support the development of potential therapeutic strategies, we established a reverse genetics system using fosmid library technology. With this system, we successfully generated recombinant PRV strains with genetic modifications, such as the expression of enhanced green fluorescent protein (EGFP) and the knockout of multiple viral genes (*US9, gI*, and g*E*). These strains enabled us to investigate viral replication, pathogenicity, and virus–host- interactions. This research will contribute to the development of improved diagnostic tools, therapeutic interventions, and vaccines for PRV, ultimately enhancing disease control strategies in the swine industry.

## 2. Materials and Methods

### 2.1. Ethics Statement

This research was carried out following the guidelines specified in the Regulations for the Administration of Affairs Concerning Experimental Animals by China’s Ministry of Science and Technology. The study protocol was approved by the Scientific Ethics Committee of Jiangsu Agri-animal Husbandry Vocational College (Permit number: JSAHVC-2024-69).

### 2.2. Virus Strain and Cell Culture

The PRV strain was isolated during a pseudorabies outbreak in Heilongjiang, China, in 2021, which affected a commercial pig farm. Infected piglets, primarily 3–5 weeks old, exhibited clinical signs such as fever, ataxia, tremors, and high mortality rates reaching up to 60% in some herds. Despite routine immunization with a commercial Bartha-K61 vaccine, the outbreak still occurred, indicating a potential vaccine escape. The isolated virus was preserved at −70 °C for further analysis. The PRV Bartha-K61 strain was obtained from the China General Microbiological Culture Collection Center (CGMCC, Beijing, China). The PRV strain was propagated using the Vero cell line. Vero cells were obtained from the China Center for Type Culture Collection (CCTCC, Wuhan, China) and grown at 37 °C in a humidified atmosphere containing 5% CO_2_. The cells were cultured in Dulbecco’s Modified Eagle Medium (DMEM, Gibco, Waltham, MA, USA) with an addition of 10% fetal bovine serum (FBS, Gemini Bio-Products, West Sacramento, CA, USA).

### 2.3. Isolation of the Virus

Diseased piglets were necropsied, and brain tissue was collected as viral material. The tissue was minced and thoroughly ground using a mortar and pestle. A certain amount of PBS was added, followed by three freeze-thaw-cycles. The homogenate was then centrifuged at 4000 rpm for 15 min, and the supernatant was collected. It was filtered through a 0.22 µm membrane to remove bacteria, resulting in a filtrate. The filtrate was immediately inoculated into cells for virus isolation. The virus isolation process was as follows: the PRV was isolated by inoculating the filtered tissue homogenate onto Vero cell monolayers, followed by incubation and observation for cytopathic effects (CPEs). After the virus induced CPEs, the supernatant was collected and subjected to freeze-thaw-cycles, serial dilution, and passaging in 96-well plates. The supernatant from the highest dilution showing CPEs was subjected to multiple rounds of plaque purification. After seven rounds of purification, the final virus was obtained and designated PRV-HL-2021.

### 2.4. PCR Identification of the Virus

The TCID_50_ was determined using the Reed–Muench method. Viral DNA was extracted and confirmed by PCR for the gB and gE genes using the specific primers listed in Table 1. The DNA of the PRV-HL-2021 strain was extracted using the TIANamp Genomic DNA kit (TIANGEN, Beijing, China) and confirmed by PCR for the gB and gE genes using the specific primers listed in Table 1.

### 2.5. Phylogenetic Analysis

Genomic DNA of the PRV-HL-2021 strain was extracted from infected cells and sent to a commercial sequencing service for next-generation sequencing and sequence assembly. Phylogenetic tree analysis, based on the complete genomic nucleotide sequences, was conducted using MEGA 7.0 software. Details of all strains included in the phylogenetic analysis are provided in Table 2.

### 2.6. Pathogenicity Analysis of PRV-HL-2021 in Mice

Thirty healthy, specific-pathogen-free BALB/c mice (6 weeks old, female) were used for the pathogenicity assays. Mice were randomly assigned to experimental groups (Groups 1–5) and a control group (Group 6). Mice in Groups 1–5 were intranasally inoculated with 100 µL of different doses (10^1^–10^5^ TCID_50_/mL) of the PRV-HL-2021 isolate through intraperitoneal administration. As a negative control, Group 6 was inoculated with 100 µL of DMEM. Clinical indicators such as depression, conjunctivitis, and scratching behavior were recorded daily, starting from the day of inoculation. A clinical scoring system ranging from normal to severe was used to quantify disease severity. The mortality rate and 50% lethal dose (LD50) in mice were calculated and used as indicators of virulence.

Mice that died during the experiment were immediately necropsied. First, viral infection in the tissues and organs was assessed by RT-PCR. Tissue and organ samples were then fixed in 4% buffered formalin, embedded in paraffin, sectioned, and stained with hematoxylin and eosin for microscopic examination.

### 2.7. Establishment of a Reverse Genetics Platform for the PRV-HL-2021 Strain

The pseudorabies virus genome was extracted from Vero cells inoculated with PRV-HL-2021. When 90% of the cells exhibited CPEs, they were collected and lysed with LCM solution and Freon. The supernatant was centrifuged, and the viral genome was released using TEN solution and SDS, followed by extraction with phenol/chloroform/isopropanol. The genomic DNA was then precipitated and dissolved in deionized water. The genome DNA was sheared, repaired, and then subjected to electrophoresis to recover fragments of 36–48 kb. These fragments were ligated into pCC1FOSTM to create recombinant plasmids, which were transduced into EPI300 cells. Monoclonal colonies were selected for sequencing, generating a fosmid library covering the full PRV genome. From 200 fosmids with foreign inserts, 19 were selected for construction. Twelve unique fosmid groups were created, designed to overlap for genomic coverage. These groups were transfected into Vero cells, with CPE producing time and viral titers recorded. Transfected Vero cells were subjected to freeze-thaw-cycles to harvest the rescued virus, named rPRV-HL-2021 [24].

### 2.8. IFA Test

The rPRV-HL-2021 was introduced into Vero cells at 90% confluence. After 48 h, the cells were fixed with cold anhydrous ethanol, treated with mouse anti-PRV antibody, washed, and then stained with Alexa 555-conjugated anti-mouse IgG.

### 2.9. Examination of the Replication Kinetics of rPRV-HL-2021

Vero cells were exposed to the virus at a MOI of 10 and kept on ice for 1 h to facilitate viral adsorption. After replacing the inoculum with pre-warmed fresh medium, cells were incubated at 37 °C for 1 h, followed by treatment with citrate buffer (pH 3.0) to inactivate the un-adsorbed virus. Cells were maintained in a fresh medium at 37 °C and 5% CO_2_, and samples were gathered at different time points of post-infection. The Reed-Muench method was used to measure viral titers in duplicate, and growth curves were created to show the phases of viral replication [24].

### 2.10. Plaque Assays

The rPRV-PRV-HL-2021 and PRV-HL-2021 were diluted in a series using DMEM, and 100 µL of each dilution was added to Vero cell monolayers. After adsorbing for one hour at 37 °C, the cells were washed with DMEM and then layered with DMEM containing 1% low-melting-point agarose. After incubating the plates at 37 °C for 4 days, plaques were stained and counted to calculate the number of plaque-forming units. These methods collectively provide detailed insights into the replication cycle and infectivity of the virus.

### 2.11. Generation of a Recombinant PRV Expressing EGFP

**Figure 1:**
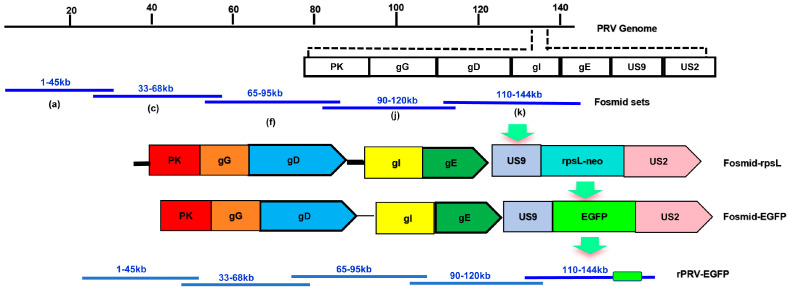
Schematic representation of the generation of recombinant PRV expressing the enhanced green fluorescent protein (EGFP) fusion protein. An EGFP expression cassette was seamlessly inserted after the stop codon of the *US9* gene via Red/ET-mediated recombination. For fosmid modification, an antibiotic-selectable cassette (rpsL-neo) flanked by two oligonucleotide homology arms was inserted after the stop codon of the *US9* gene. The EGFP gene, also flanked by the same homology arms, replaced the rpsL-neo cassette to generate Fosmid-EGFP. For whole genome assembly, Fosmid-EGFP and other fosmids were transfected into Vero cells to produce a recombinant PRV expressing the EGFP gene.

The EGFP gene was inserted after the *US9* gene using a Counter Selection BAC Modification Kit (Gene Bridges, Berkeley, CA, USA) according to the manufacturer’s instructions. Initially, the Red/ET expression plasmid (pRed/ET) and the fosmid were introduced into competent *E. coli* DH10B cells using electroporation. Subsequently, the antibiotic resistance cassette (rpsL-neo), bordered by homology arms, was created via PCR amplification, with specific primers detailed in Table 2. The cassette was then inserted into the target region of the fosmid using Red/ET recombination. Electrocompetent cells were created from *E. coli* cells with the modified fosmid containing the rpsL-neo cassette to facilitate EGFP gene expression. Before this step, the EGFP gene and its surrounding homology arms were amplified using specific primers listed in Table 1. This EGFP segment was then inserted into electrocompetent cells, substituting the rpsL-neo cassette via Red/ET recombination. The construction strategy is illustrated in Figure 1. Ultimately, the modified fosmid, together with the required fosmids, was introduced into Vero cells to rescue the recombinant virus. Vero cells transfected with a collection of fosmids lacking one fosmid acted as a negative control (Figure 1).

### 2.12. Generation of a Recombinant PRV with Three Gene Knockouts

**Figure 2:**
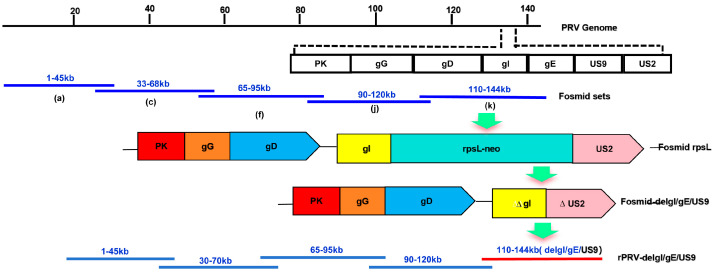
**Schematic diagrams of the PRV recombinants.** The schematic representation of the fosmid in which partial coding regions of glycoprotein I (gI), glycoprotein E (gE), and *US9* genes are deleted. For fosmid modification, the antibiotic-selectable cassette (rpsL-neo) flanked by two oligonucleotide homology arms was inserted between the second acids of *gI* and third amino of *US2*. The partial coding regions of glycoprotein I (*gI*) and *US2* flanked by the same homology arms were used to replace the rpsL-neo cassette to generate Fosmid-del*gI/gE/US9*. For whole genome assembly, Fosmid-del*gI/gE/US9* and other fosmids were transfected into Vero cells to generate a recombinant PRV, in which partial coding regions of *gI* and *US2* and whole *gE* and *US9* genes are deleted.

Using Red/ET recombination technology, a fosmid that knocks out the *US9*, *gI*, and *gE* genes was modified through a two-step process: (1) a selection marker gene rpsL-neo expression cassette with homologous arms was inserted into the corresponding position of the fosmid; (2) the selection marker (rpsL- neo) was replaced with portions of the *US2* and *gI* genes of Bartha K61 flanked by the same 50 bp homologous arms. Positive clones were screened on LB plates containing chloramphenicol and streptomycin, and further identified by kanamycin resistance selection and PCR. The construction strategy is illustrated in Figure 2. The modified fosmids (*US9*, *gI*, and *gE* knockouts) were co-transfected with four additional fosmids into Vero cells. The negative control group was transfected with only four fosmids. After observing CPEs in the transfected cells, the mixture of cells and culture supernatant was collected and subjected to several freeze-thaw-cycles to extract the rescued virus, named rPRV-del*gI/gE/US9* (a strain with three gene deletions).

## 3. Results

### 3.1. Isolation and Identification of PRV Epidemic Strain

**Figure 3:**
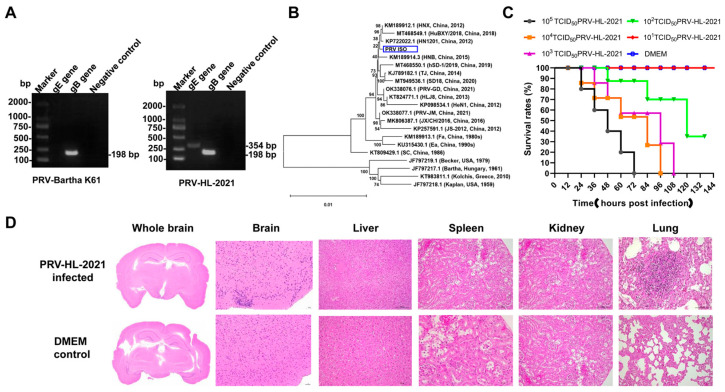
(**A**) PCR identification of PRV-HL-2021 and PRV-Bartha K61. Viral DNA of PRV-HL-2021 and PRV-Bartha K61 was extracted, and PRV *gE* and *gB* genes were amplified. (**B**) Phylogenetic tree based on complete genome nucleotide sequences. (**C**) Pathogenicity of PRV strain in mice. Thirty healthy 6-week-old mice were randomly divided into 6 groups (5 mice per group). Groups 1–5 were intranasally inoculated with 100 µL of different doses (10^1^ TCID_50–_10^5^ TCID_50_) of the PRV-HL-2021 isolates, respectively. Group 6 served as the negative control, receiving 100 µL of DMEM. (**D**) Histopathological lesions of brain, liver, spleen, kidney, and lung from euthanized mice. Original gel images, Figure S1.

After inoculating the filtered supernatant from the viral material into Vero cells and blind-passaging for three generations, typical CPEs were observed in the Vero cells. Following six cycles of plaque purification and PCR identification, an isolated PRV called PRV-HL-2021 was received. The DNA of PRV-HL-2021 was extracted, and PCR amplification was performed. The amplification products were sent for sequencing, and the presence of two specific bands at 325 bp (*gB*) and 198 bp (*gE*) confirmed that PRV-HL-2021 is the currently circulating wild strain (Figure 3A and Figure S1).

To investigate the genetic evolutionary relationships of the PRV-HL-2021 strain, phylogenetic trees were constructed based on complete genomic nucleotide sequences (Figure 3B). The analysis revealed that the PRV-HL-2021 strain was closely related to Chinese isolates, distinguishing it from foreign representative strains such as Kaplan, Kolchis, Bartha, and Becker. Furthermore, PRV-HL-2021 was more closely related to the Chinese isolates of HN11201, which were collected after 2011, than to earlier Chinese isolates of representative strains like SC, Ea, and Fa.

### 3.2. The Pathogenicity of PRV-HL-2021

To evaluate the pathogenicity of PRV-HL-2021, experimental infections were conducted using mice. Observations of the mice’s behavior, feeding, and clinical symptoms were recorded. Throughout the experiments, all mice that were mock-infected stayed healthy. When infected with doses exceeding 10^2^ TCID_50_ of PRV-HL-2021, mice exhibited intense itching from 1 dpi and died between 1 and 5 dpi (Figure 3C). Through calculation with the Reed-Muench method, LD50 of PRV-HL-2021 was 10^2.167^ TCID_50_, indicating that the PRV-HL-2021 strain was the more virulent strain for mice. Quantitative PCR analysis confirmed the presence of PRV DNA in both brain and heart tissue samples, indicating systemic viral dissemination beyond the primary sites of infection. The detection of viral genetic material in these tissues suggests a potential tropism of PRV for neural and cardiac systems, which may contribute to disease pathogenesis and clinical manifestations (Table 3). Histopathological examination of hematoxylin and eosin (HE)-stained tissue sections revealed mild perivascular and parenchymal inflammatory infiltrates in the brain and lungs, consistent with a localized immune response to viral infection. In contrast, no significant histological lesions, such as necrosis or hemorrhage, were observed in the liver, kidney, spleen, or gastrointestinal tract, indicating that PRV-associated tissue damage was largely restricted to the central nervous and respiratory systems (Figure 3D).

### 3.3. Construction of a PRV Reverse Genetics Platform Based on the Fosmid Library

**Figure 4:**
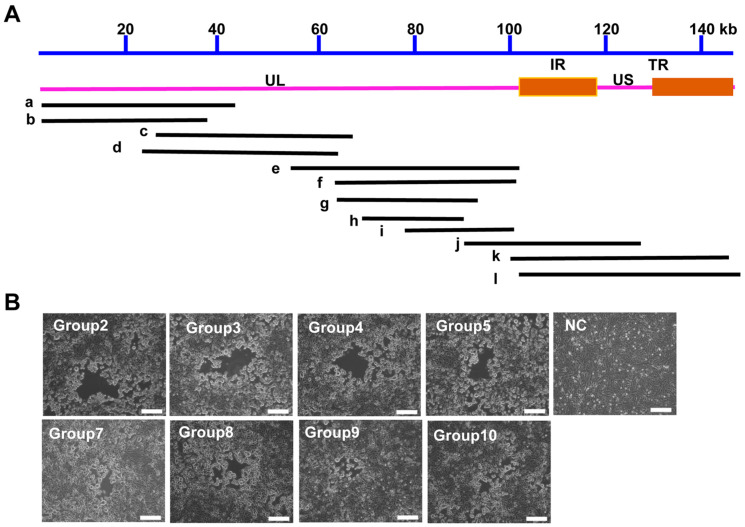
(**A**) **Relative location of 5 selected overlap fosmids in the PRV-HL-2021 strain genome.** Monoclonal colonies were selected for sequencing, generating a fosmid library that covers the full PRV genome. In total, 12 fosmids (named as PRV-IF-a, b, c, d, e……l) were selected from a pool of 200 fosmids with foreign inserts for construction. (**B**) **CPE of rescued PRV-HL-2021 (rPRV-HL-2021) in Vero cells.** Scale bar = 100 μm. Twelve unique fosmid groups, designed for genomic coverage, were transfected into Vero cells. Eight fosmid groups produced CPEs after transfection.

From the fosmid library, 200 clones were randomly picked for end sequencing, achieving more than double coverage of the PRV genome. The end sequencing results indicated that these fosmids cover the entire PRV genome. However, there was a relative abundance of fosmids containing UL region fragments, suggesting a certain bias in the constructed fosmid library. Most fosmids had inserts ranging from 30 to 40 kb; 12 fosmids were chosen to create combinations that together span the entire PRV genome (Table 4). For the rescue of recombinant PRV, 10 sets of overlapping fosmids were transfected to the Vero cells (Table 5 and Figure 4A). Among these groups, eight groups rescued the virus successfully, and the rescued virus was named rPRV-HL-2021 (Figure 4B).

**Figure 5:**
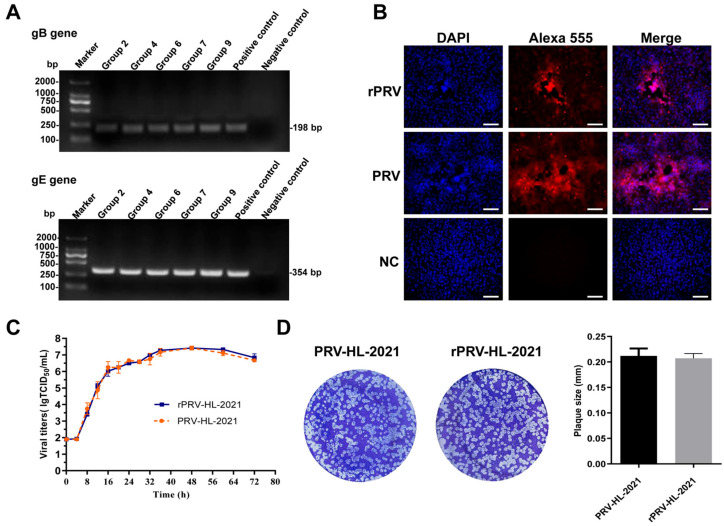
(**A**) **PCR identification of rPRV-HL-2021.** The *gB* and *gE* genes were amplified using the genome of the rescued virus as a template. The parental PRV genome served as a positive control, and an irrelevant genome was used as a negative control. (**B**) **IFA identification of rPRV-HL-2021 in Vero cells.** rPRV-HL-2021 was detected by IFA using anti-PRV serum. (**C**) **Replication kinetics of rPRV-HL-2021 in Vero cells.** Scale bar = 100 μm. Vero cells were infected with the virus at a MOI of 10, incubated on ice for 1 h to allow viral adsorption, and then the inoculum was replaced with fresh medium. Cells were incubated at 37 °C for 1 h, followed by treatment with citrate buffer (pH 3.0). Samples were collected at various post-infection time points. Viral titers were determined using the Reed–-Muench method. (**D**) **Plaque assays of rPRV-HL-2021**. For plaque assays, rPRV-HL-2021 and PRV-HL-2021 were serially diluted in DMEM, added to Vero cell monolayers, incubated at 37 °C, washed, and then overlaid with DMEM containing 1% low-melting-point agarose. Plates were incubated for 5 days at 37 °C, after which plaques were stained and counted to calculate plaque-forming units. Original gel images, Figure S2.

After being rescued, rPRV-HL-2021 experienced several freeze-thaw-cycles and was continuously passaged for ten generations in Vero cells. DNA was extracted from the strain at the 10th generation, and PCR amplification of the gB and gE fragments was performed. Figure 5A shows that PRV *gE* and *gB* genes were successfully detected (Figure S2). The immunofluorescence assay (IFA) results confirmed the successful rescue of the recombinant virus. Infected cells exhibited specific red fluorescence when probed with PRV-infected serum, while uninfected control cells showed no detectable fluorescence (Figure 5B). The growth curve of the rescued virus is comparable to that of the PRV-HL-2021 strain, demonstrating that the growth characteristics of the two viruses are consistent (Figure 5C).

### 3.4. Rescue and Characterization of Recombinant Virus Expressing EGFP

**Figure 6:**
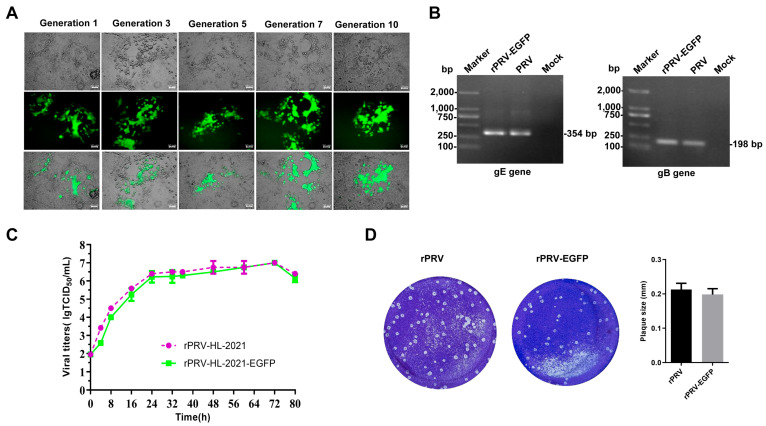
(**A**) **CPE of rescued rPRV-EGFP in Vero cells.** The recombinant virus (rPRV-EGFP) expressing EGFP was rescued. After serial passage for 10 generations, the EGFP gene remains stably expressed. (**B**) **PCR identification rPRV-EGFP**. The gB and gE genes were amplified using the genome of rPRV-EGFP as a template. The parental PRV genome served as a positive control, and an irrelevant genome was used as a negative control. (**C**) **Replication kinetics of rPRV-EGFP in Vero cells.** Scale bar = 50 μm (**D**) **Plaque assays o rPRV-EGFP**. Original gel images, Figure S3.

The modified fosmid (which incorporates an EGFP expression cassette), when co-transfected with other fosmids into Vero cells, can generate PRV expressing EGFP. The rescued virus rPRV-US9-EGFPE is capable of inducing CPEs. After serial passage of rPRV-US9-EGFP for 10 generations, the EGFP gene remained stably expressed, and the gB and gE genes were stable in the rPRV-US9-EGFPE (Figure 6A,B and Figure S3). The growth curve and the plaque of rPRV-US9-EGFP is comparable to that of the PRV-HL-2021 strain, demonstrating that the growth characteristics of the two viruses are consistent (Figure 6C,D).

### 3.5. Rescue of the Recombinant Virus Deleting gI/gE/US9 Genes

**Figure 7:**
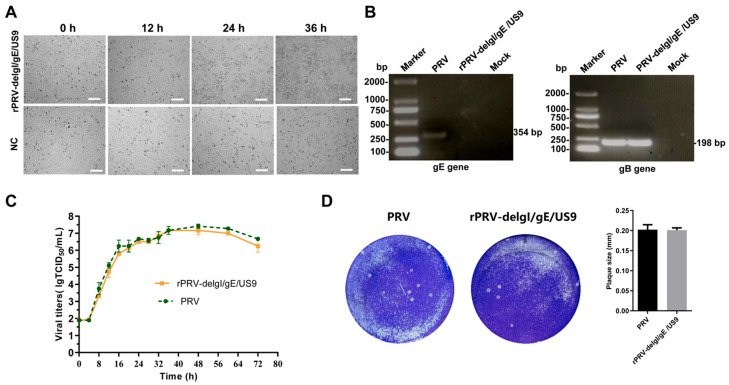
(**A**) **CPE of rescued three-gene-deleted PRV-HL-2021 (rPRV-del*gI/gE/US9*) in Vero cells.** Scale bar = 200 μm. The modified fosmids (with *US9, gI,* and *gE* knockouts) were co-transfected with four additional fosmids into Vero cell plates. The negative control group used only the four fosmids, which did not produce CPE. (**B**) **PCR identification of rPRV-del*gI/gE/US9*.** PRV *gE* and *gB* genes were amplified using the genome of rPRV-del*gI/gE/US9* as a template. The parental PRV genome served as a positive control, and an irrelevant genome was used as a negative control. (**C**) **Replication kinetics of rPRV-del*gI/gE/US9* in Vero cells.** (**D**) **Plaque assays o rPRV-del*gI/gE/US9***. Original gel images, Figure S4.

After co-transfecting the fosmid knocking out *gI, gE,* and *US9* with the other four fosmids into Vero cells for 24 h, significant CPEs were observed (Figure 7A). This three-gene-deleted recombinant was named rPRV-delgI/gE/US9. The rPRV-delgI/gE/US9 virus was subjected to multiple freeze-thaw-cycles and passaged ten times in Vero cells. DNA was extracted from this strain at the 10th generation, and gB and gE fragments were amplified using the primer pairs, as described earlier. Effective amplification of gB was observed, while no amplification of the gE fragment occurred (Figure 7B and Figure S4).

## 4. Discussion

Pseudorabies virus (PRV), a member of the Herpesviridae family, remains a critical threat to the swine industry worldwide due to its highly contagious nature and severe impact on animal health. While it predominantly affects swine, PRV has demonstrated the ability to cross species barriers and infect a variety of mammals, including humans, under certain circumstances [25,26]. Although human infections remain rare, they raise serious concerns due to the zoonotic potential of the virus and the possibility of future pandemics. Recent studies, including the case of human encephalitis in China caused by a new PRV variant, have underscored the importance of understanding the full range of PRV’s potential impact on public health [27]. The cross-species transmission of PRV, particularly in regions with frequent human-wildlife-livestock interactions, makes it critical for public health systems to be vigilant in monitoring the virus’s spread and potential for wider infection in non-swine species [28].

The PRV-HL-2021 strain isolated in this study provides valuable insights into the genetic diversity and pathogenicity of circulating PRV variants in China. Compared to earlier Chinese strains, it shares several virulence-associated mutations in key glycoprotein genes that may contribute to its heightened pathogenicity. In mouse models, PRV-HL-2021 caused rapid onset of severe neurological and respiratory symptoms, consistent with or exceeding the virulence observed in these previously characterized strains [29]. These findings further reinforce the need for targeted surveillance and rapid response measures to contain potential outbreaks. By understanding the genetic profile and behavior of circulating PRV strains, especially those with heightened virulence, it becomes possible to better anticipate and mitigate the risks posed by future epidemics, including those involving zoonotic spillover events.

The construction of a high-quality PRV fosmid library and the establishment of a reverse genetics system require meticulous optimization of DNA handling, cloning strategies, and screening methods [30]. Key challenges-such as DNA shearing, incomplete genome coverage, and inefficient viral rescue-must be addressed to enhance the robustness of PRV genetic manipulation. PRV possesses a large (~150 kb), double-stranded DNA genome, which is highly susceptible to shearing during extraction and handling, often resulting in incomplete or truncated clones. To mitigate this, we employed gentle DNA extraction protocols to preserve genomic integrity and optimized partial digestion conditions to generate fragments suitable for fosmid cloning. To ensure fragment sizes of approximately 40–45 kb, which is ideal for fosmid vectors, we utilized pulsed-field gel electrophoresis (PFGE) for precise size selection. Despite these measures, the resulting PRV fosmid library exhibited a positional bias-clones predominantly represented sequences from the central region of the genome, with fewer fosmids covering terminal regions. To address this imbalance, we designed overlapping primer sets spanning the entire PRV genome and implemented multiplex PCR for efficient, high-throughput clone screening across multiple genomic regions. Additionally, transfection of the large PRV genome into mammalian cells often yields low infectivity, potentially due to the DNA size or suboptimal assembly. To improve transfection efficiency and successful viral rescue, we optimized both transfection conditions and cell line selection. Specifically, we found that doubling the volume of transfection reagent and using Vero cells significantly enhanced virus recovery efficiency.

A key contribution of this study is the establishment of a reverse genetic platform based on the fosmid library for PRV-HL-2021. The ability to manipulate the PRV genome using this system represents a significant advancement in the field of viral genetics. The fosmid-based reverse genetics system provides several advantages, including a more efficient and higher capacity approach to generating recombinant PRV strains compared to traditional methods. This platform facilitates the precise modification of the PRV genome, enabling researchers to create viral strains with specific genetic alterations. For instance, the generation of the EGFP-expressing recombinant virus (rPRV-*US9*-EGFP) allows for real-time tracking of viral replication and host-virus-interactions, offering valuable insights into the dynamics of infection at the cellular level.

Equally important is the development of recombinant PRV strains with targeted gene deletions, such as *US9, gI*, and *gE*, as demonstrated in the rPRV-del*gI/gE/US9* strain. These deletions have been shown in previous studies to attenuate virulence while preserving immunogenicity, making them promising candidates for live-attenuated vaccines [30]. Our findings support this approach, as the modified virus maintains replication competence in vitro. This replication competence is critical for vaccine development, particularly in addressing the limitations of current vaccines like Bartha-K61, which have shown reduced protection against emerging PRV variants in China [31,32,33]. The reverse genetics platform we established not only enables precise gene editing for rational vaccine design but also facilitates rapid screening of antigenic candidates and antiviral targets. Overall, these tools are essential for enhancing PRV control strategies in the field and reducing the threat of cross-species transmission.

As indicated by our current experimental results, the growth curve and plaque morphology of the rescued virus are comparable to those of the parental PRV-HL-2021 strain, indicating similar replication kinetics and in vitro growth characteristics. These findings suggest that there are no significant differences in replication capacity under the tested conditions. However, we acknowledge the importance of further investigating potential differences in pathogenicity and underlying molecular mechanisms. Future studies involving *in vivo* infection models and transcriptomic or proteomic analyses are planned to comprehensively assess the biological properties of the recombinant viruses compared to the parental strain [30].

This study highlights the broader significance of reverse genetics platforms in addressing infectious diseases like PRV. By generating recombinant viruses that can be studied *in vivo*, this approach offers a powerful tool for understanding viral mechanisms of action, host immune responses, and potential therapeutic interventions. The flexibility of the platform, demonstrated by the generation of recombinant PRV strains with varying genetic modifications, will be instrumental in future research aimed at improving diagnostic tools, developing antiviral drugs, and enhancing vaccine strategies. The ability to tailor viral strains for specific research purposes is an essential step toward creating more effective and targeted solutions for controlling PRV and other viral infections.

## 5. Conclusions

In conclusion, the successful isolation and characterization of the PRV-HL-2021 strain, together with the establishment of a reverse genetics system, represent significant progress in understanding and combating PRV infections. These advancements provide powerful tools for dissecting viral pathogenesis, developing next-generation vaccines and antiviral therapies, and improving diagnostic approaches, thereby contributing to more effective disease control strategies. However, several limitations should be acknowledged. During the virus isolation process, potential risks of contamination remain a challenge, and while strict biosafety and purification protocols were applied, further refinement of these methods would enhance the reliability of future isolations. Additionally, although the reverse genetics system was successfully employed to generate recombinant viruses, we encountered notable challenges in efficiency, particularly during genome segment assembly and virus rescue. Future efforts should focus on optimizing transfection conditions and improving vector design to enhance the robustness of this system. In the future, further research is needed to investigate the mechanisms underlying PRV’s zoonotic transmission and its interaction with host species at the molecular level. In addition to vaccine development, attention should also be directed toward understanding PRV coinfections with other pathogens, which may influence disease severity and epidemiology. Moreover, the reverse genetics platform established in this study offers a valuable tool not only for studying viral gene functions but also for high-throughput screening of antiviral compounds. Collectively, these directions will not only broaden our understanding of PRV biology and its public health implications but also pave the way for translational applications in both veterinary and potentially human medicine.

## Figures and Tables

**Table 1:** Primers used in this study.

No.	Name of the Primer	Sequences of the Primers	Target Genes
1	F-identify-gE	TGGCTCTGCGTGCTGTGCTC	gE
	R-identify-gE	CATTCGTCACTTCCGGTTTC	
2	F-identify- gB	GGGGTTGGACAGGAAGGACACCA	gB
	R-identify- gB	AACCAGCTGCACGCGCTCAA	
3	F-US9-rpsL	TCTGCTCGCTGTCCGCGCTACTCGGGGGCATCGTCGCCAGGCACGTGTAGGCTCCCCGCGGGGCTCCTCC	rpsL
	R-US9-rpsL	GGGCGCGGCGGATGGGGGCGGGCCCCCGCTCCCGTTCGCTCGCTCGCTCGCCTCGGCGCCGGCGCACGTC	
4	F-replace rpsL-insert-EGFP	TCTGCTCGCTGTCCGCGCTACTCGGGGGCATCGTCGCCAGGCACGTGTAGTAGTTATTAATAGTAATC	EGFP
	R-replace rpsL-insert-EGFP	GGGCGCGGCGGATGGGGGCGGGCCCCCGCTCCCGTTCGCTCGCTCGCTCGGCAGTGAAAAAAATGCTT	
5	F-delgE/gI/US9-rpsL	GCCTCCGCAGTACCGGCGTCGATGATGATGGTGGCGCGCGACGTGACCCGGCAGTGAAAAAAATGCTT	rpsL
	R-delgE/gI/US9-rpsL	TCTAGGAGATGGTACATCGCGGGGCGCGCTCGCGTCCGTTGCCGCGCCCGTCAGAAGAACTCGTCAAGAAGGCG	
6	F-replace rpsL-delgE/gI/US9	TCTAGGAGATGGTACATCGCGGGGCGCGCTCGCGTCCGTTGCCGCGCCCGCCTCGGCGCCGGCGCACGTC	Parts of US2 and US7

**Table 2:** Reference PRV strains used for phylogenetic analysis.

PRV Strain	Country	Year of Isolating	GenBank No.
HNX	China	2012	KM189912.1
HuBXY/2018	China	2018	MT468549.1
HN1201	China	2012	KP722022.1
HNB	China	2015	KM189914.1
hSD-1/2019	China	2019	MT468550.1
TJ	China	2014	KJ789182.1
SD18	China	2020	MT949536.1
PRV-GD	China	2021	OK338076.1
HLJ8	China	2013	KT824771.1
HeN1	China	2012	KP098534.1
PRV-JM	China	2021	OK338077.1
JX/CH/2016	China	2016	MK806387.1
JS-2012	China	2012	KP257591.1
Fa	China	1980s	KM189913.1
Ea	China	1990s	KU315430.1
SC	China	1986	KT809429.1
Becker	USA	1979	JF797219.1
Bartha	Hungary	1961	JF797217.1
Kolchis	Greece	2010	KT983811.1
Kaplan	USA	1959	JF797218.1

**Table 3:** Real-time PCR was used to detect the distribution of the virus in each tissue.

		The Number of Animals That Can Detect PRV						
Strain	Dose (TICD50)	Route	Brain	Heart	Liver	Spleen	Lung	Kidney
PRV-HL-2021	10^5^	i.p.	4/5	1/5	0/5	0/5	0/5	0/5
	10^4^	i.p.	2/5	0/5	0/5	0/5	0/5	0/5
	10^3^	i.p.	2/5	0/5	0/5	0/5	0/5	0/5
	10^2^	i.p.	2/5	0/5	0/5	0/5	0/5	0/5
DMEM	0.1 mL	i.p.	0/5	0/5	0/5	0/5	0/5	0/5

**Table 4:** The size of the fragment inserted in each fosmid and its relative position in the genome of the PRV-HL-2021 strain.

Fosmid	Relative Position in the Genome (nt)	The Size of the Fragment (bp)	Fosmid	Relative Position in the Genome (nt)	The Size of the Fragment (bp)
PRV-IF-a	1–44102	44,101	PRV-IF-g	50226–89728	39,502
PRV-IF-b	1–39716	39,515	PRV-IF-h	51898–85801	33,903
PRV-IF-c	33891–68519	34,628	PRV-IF-i	67889–102713	34,824
PRV-IF-d	28749–61624	32,875	PRV-IF-j	91337–122601	25,883
PRV-IF-e	47680–10987	35,793	PRV-IF-k	100865–end	26,640
PRV-IF-f	61229–101922	40,693	PRV-IF-l	101794–end	25,711

**Table 5:** Fosmid combinations that rescue viruses.

Group	Fosmid Combination	CPE Occurrence Time (hpt)
1	a + c + e + j + k	no CPE occurrence
2	a + c + f + j + k	24
3	b + d + f + g + h + i	48
4	b + d + h + i + k	36
5	b + d + h + i + j + k	54
6	a + c + e + j + l	no CPE occurrence
7	a + c + f + j + l	36
8	b + d + f + g + h + l	54
9	b + d + h + i + l	36
10	b + d + h + i + j + l	36

## Data Availability

All data are contained within this article and its Supplementary Materials.

## Supplementary Materials

The following supporting information can be downloaded at: https://www.mdpi.com/article/10.3390/vetsci12060519/s1, Figure S1: The original gel images of Figure 3A. Figure S2:The original gel images of Figure 5A. Figure S3: The original gel images of Figure 6B. Figure S4: The original gel images of Figure 7B.
