# Supplementary material for: Identification, Pathogenicity, and Reverse Genetics System Construction of a Pseudorabies Virus Isolate from Pigs in China
Source: Vet Sci. 2025 May 26;12(6):519. doi: 10.3390/vetsci12060519 (PMC12197798; doi:10.3390/vetsci12060519)
Supplement: Supplementary file 1 [file vetsci-12-00519-s001.zip › vetsci-3586923-supplementary.pptx]

## Slide 1
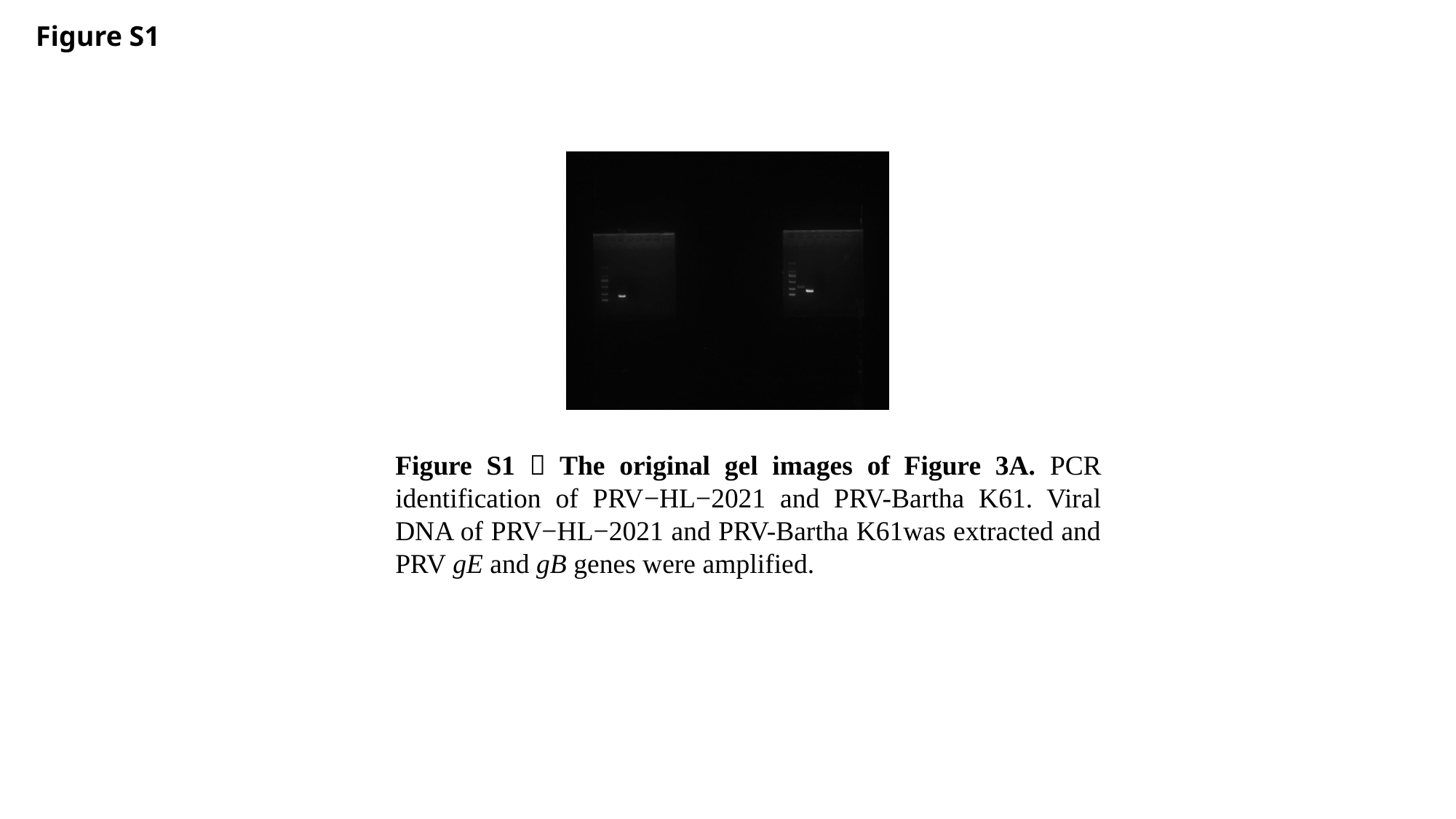

Figure S1
Figure S1：The original gel images of Figure 3A. PCR identification of PRV−HL−2021 and PRV-Bartha K61. Viral DNA of PRV−HL−2021 and PRV-Bartha K61was extracted and PRV gE and gB genes were amplified.

## Slide 2
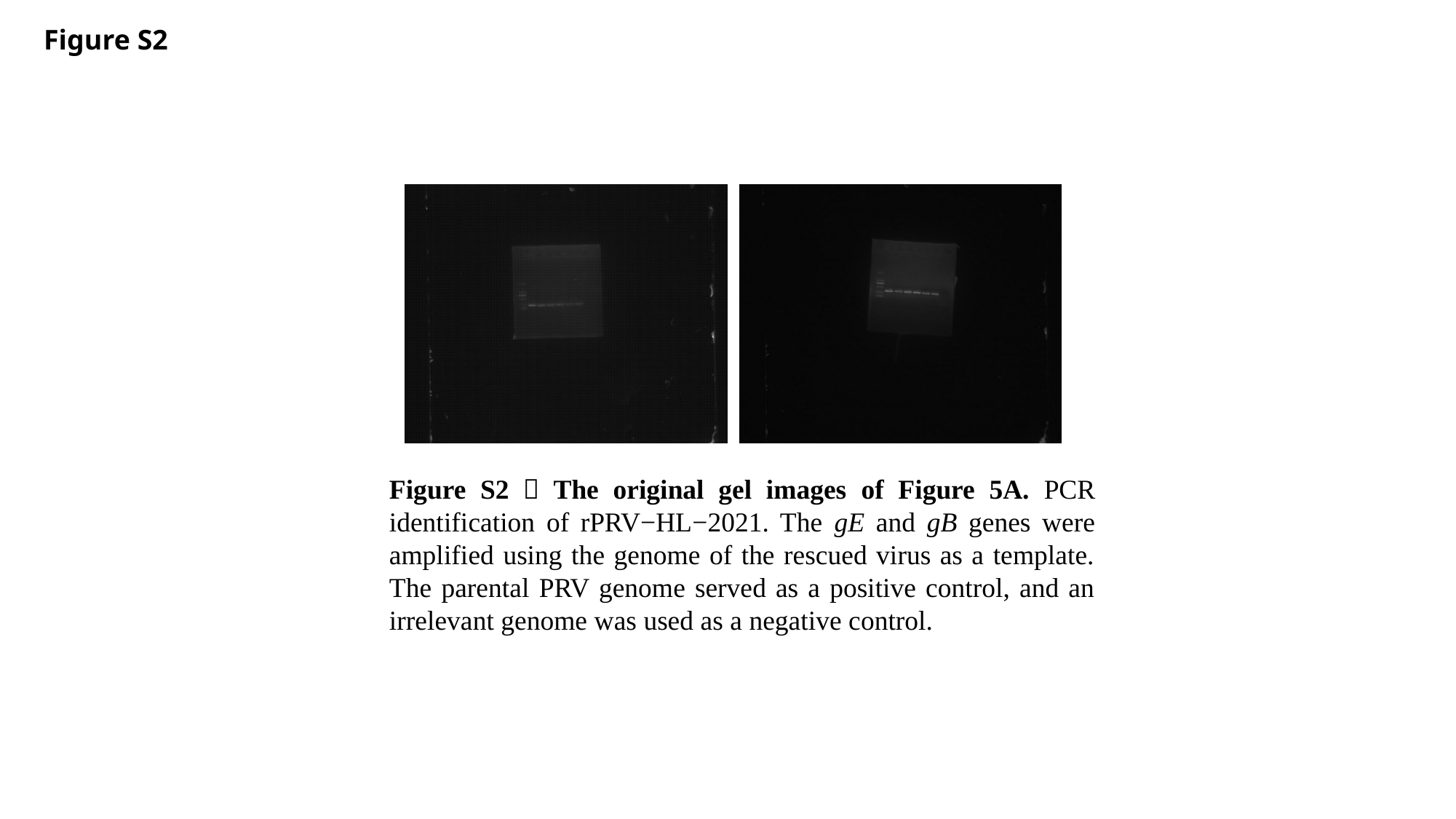

Figure S2
Figure S2：The original gel images of Figure 5A. PCR identification of rPRV−HL−2021. The gE and gB genes were amplified using the genome of the rescued virus as a template. The parental PRV genome served as a positive control, and an irrelevant genome was used as a negative control.

## Slide 3
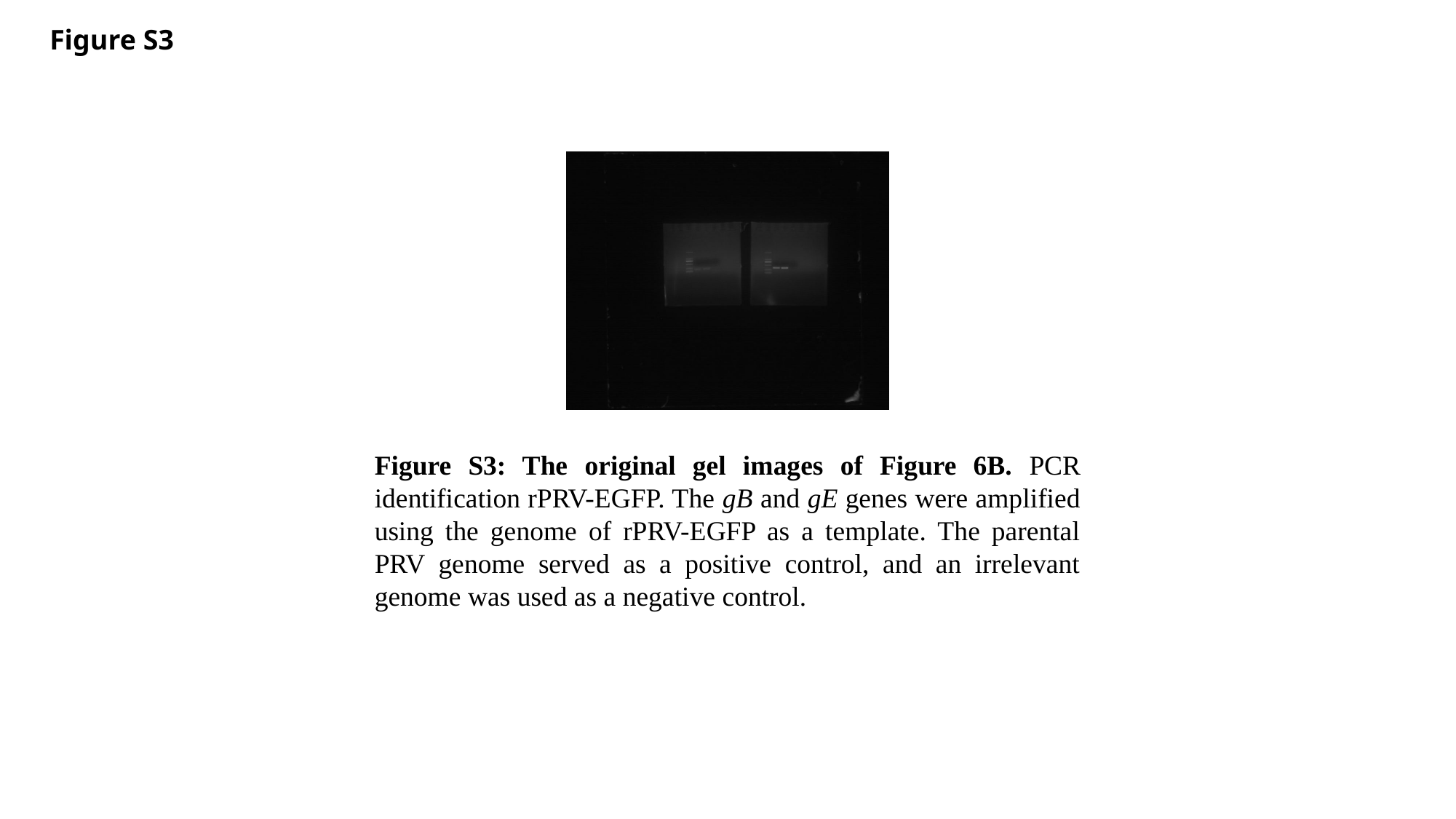

Figure S3
Figure S3: The original gel images of Figure 6B. PCR identification rPRV-EGFP. The gB and gE genes were amplified using the genome of rPRV-EGFP as a template. The parental PRV genome served as a positive control, and an irrelevant genome was used as a negative control.

## Slide 4
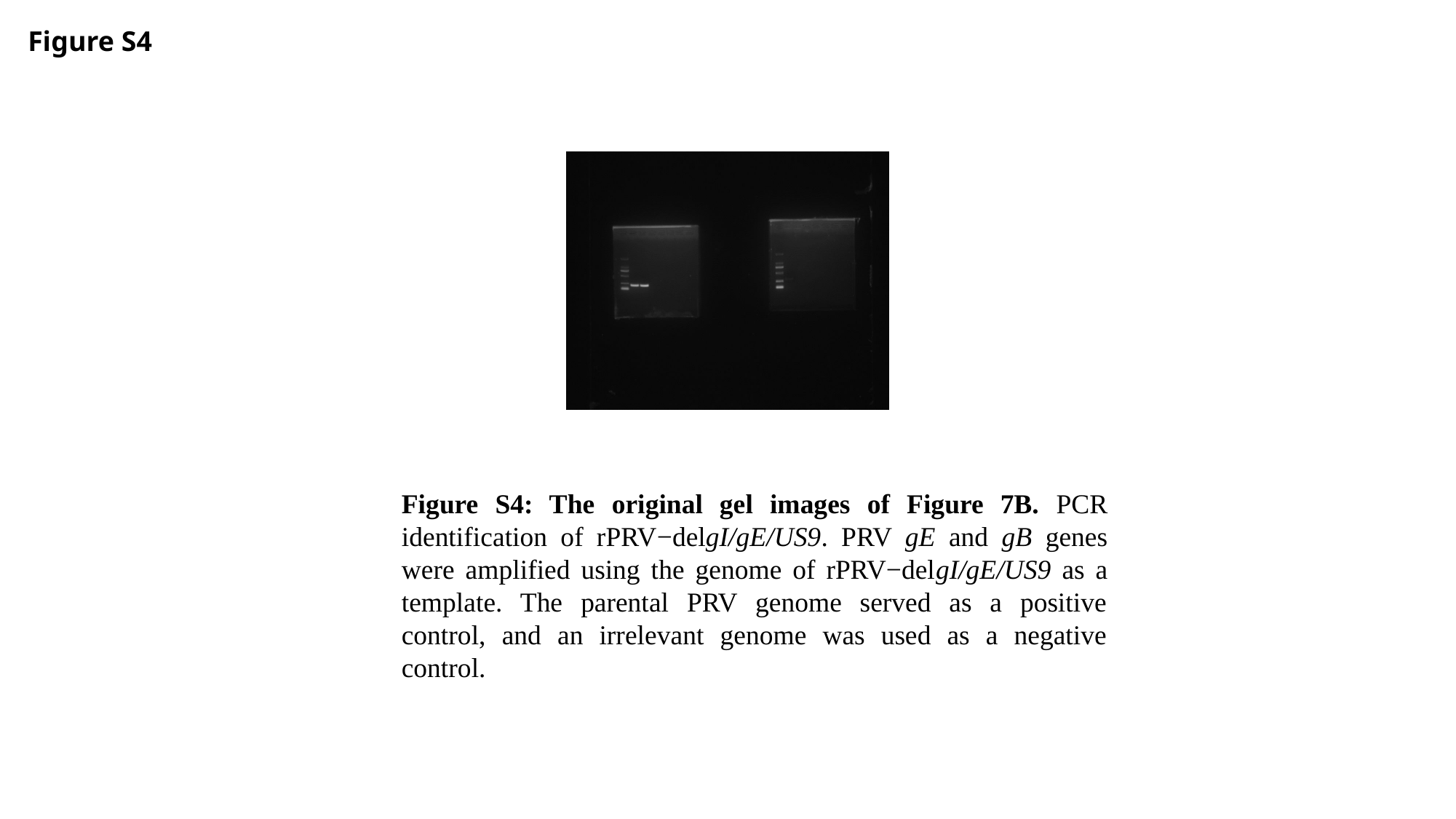

Figure S4
Figure S4: The original gel images of Figure 7B. PCR identification of rPRV−delgI/gE/US9. PRV gE and gB genes were amplified using the genome of rPRV−delgI/gE/US9 as a template. The parental PRV genome served as a positive control, and an irrelevant genome was used as a negative control.
